# Regeneration in the Segmented Annelid *Capitella teleta*

**DOI:** 10.3390/genes12111769

**Published:** 2021-11-08

**Authors:** Elaine C. Seaver, Danielle M. de Jong

**Affiliations:** Whitney Laboratory for Marine Bioscience, University of Florida, Saint Augustine, FL 32080, USA; dandej@whitney.ufl.edu

**Keywords:** annelid, *Capitella teleta*, regeneration, germline, Hox genes, multipotent progenitor cells, germline

## Abstract

The segmented worms, or annelids, are a clade within the Lophotrochozoa, one of the three bilaterian superclades. Annelids have long been models for regeneration studies due to their impressive regenerative abilities. Furthermore, the group exhibits variation in adult regeneration abilities with some species able to replace anterior segments, posterior segments, both or neither. Successful regeneration includes regrowth of complex organ systems, including the centralized nervous system, gut, musculature, nephridia and gonads. Here, regenerative capabilities of the annelid *Capitella teleta* are reviewed. *C. teleta* exhibits robust posterior regeneration and benefits from having an available sequenced genome and functional genomic tools available to study the molecular and cellular control of the regeneration response. The highly stereotypic developmental program of *C. teleta* provides opportunities to study adult regeneration and generate robust comparisons between development and regeneration.

## 1. Introduction

The ability to replace lost body parts is widespread across animals. Elucidating the cellular and molecular mechanisms that control regeneration in a range of animals and interpreting this data in a rigorous phylogenetic context will enable an understanding of the evolution of relative regenerative success. Increased interest in regeneration over the past decade and recent technical advances (e.g., genome sequencing, CRISPR/Cas9) has expanded the number of animals currently being investigated for modern regeneration studies. This review summarizes data on posterior regeneration for the annelid *Capitella teleta* and outlines its advantages for future regeneration research. Annelid regeneration is briefly introduced here to provide context, and the reader is encouraged to look at one of the numerous excellent comprehensive reviews on various aspects of annelid regeneration [1,2,3,4,5,6,7,8,9].

Annelids, the segmented worms, have long been known for their extensive regeneration abilities [2,5,6]. There is considerable variation in regenerative capacities across species. Some annelids can regenerate their heads and tails or reform the whole body from a small tissue fragment [2]. Others can regenerate only their tails and yet others have completely lost the ability to regenerate any segments. Both anterior and posterior regeneration abilities are ancestral for annelids, and there have been numerous evolutionary losses in regeneration [3]. Regeneration of complex organ systems observed in annelids likely requires coordination among multiple tissues, and annelids can be used to investigate in vivo cellular reprogramming. One unique asset that annelids bring to regeneration studies is their segmented body plan. Because segments can be counted, and there are often morphological differences between individual segments (e.g., thoracic versus abdominal), there can be precision in amputation location and tests of positional information. This provides a spatial reference over time during a highly dynamic process.

The history of annelid regeneration studies goes back over a century and there is substantial literature on a wide range of annelid species (reviewed in [1,5,6,7]). Histological investigations of fixed tissue provide evidence of local tissue responses to amputation that may be germ layer restricted and reflect coordination among multiple tissues for successful regeneration. This complex local response, which includes interactions across tissues, is reminiscent of vertebrate limb regeneration [10].

In addition to descriptive and histological work, there are classic experimental studies that address central questions in regeneration biology. For example, one set of experimental studies included X-ray irradiation of part or of the whole animal followed by amputation (reviewed in [7]). These studies addressed the question of the cellular origin of regenerating tissue, specifically of the possible migration of stem cells into the wound site from a distant location. In general, whole animal irradiation inhibits regeneration. In contrast, local irradiation of numerous segments adjacent to the amputation site resulted in successful regeneration in some species, suggesting a role for cells that originate from a distant site. Cells with a large nuclear to cytoplasmic ratio have been documented in the coelomic cavity of annelids. These cells were thought to represent a somatic stem cell population and were called neoblasts [11]. Morphological similarities between neoblasts and germ cells have been noted in annelids, specifically cells with a large nuclear to cytoplasmic ratio [12,13]. Other experimental manipulations include the demonstration of a role for the nervous system in organizing the regenerative response by transection of the ventral nerve cord near the wound site, and surgeries that repositioned the nerve cord to an ectopic position often resulted in the formation of a blastema near the nerve [5,7,14]. In addition, the ventral nerve cord may have a role in patterning the dorsal–ventral axis, as shown by dorsalization of the regenerate when regeneration occurs in the absence of the nerve cord [15].

More recent efforts have focused on beginning to build a molecular understanding of events such as wound response and cellular reprogramming during annelid regeneration. These studies have included spatial and temporal characterization of genes expressed at the wound site using both candidate gene approaches and generation of bulk transcriptome/RNA Seq datasets (reviewed in [9]). The role of molecular signaling in the regeneration response has been investigated using chemical inhibitors studies in a few cases; one such study implicates FGF signaling in the control of cell division in the regenerating tissue [16]. In addition, characterization of cell division profiles demonstrates a requirement for cell division during regeneration [17]. Functional genomic approaches have been developed for a small number of annelids and will likely be applied to regeneration studies in the coming years.

Here, we review previously published studies of regeneration in *C. teleta*, an annelid that exhibits posterior regeneration. New expression data for several genes are also presented to provide more detailed molecular characterization of the regeneration response in *C. teleta*. These include expression patterns of a gene whose upregulation in response to amputation occurs within hours, stem cell marker genes and markers of specific cell types or organs in regenerating tissue. Specifically, material presented in Figures 4, 7B–D and 8A is published for the first time here.

## 2. Materials and Methods

A *C. teleta* colony was maintained in the laboratory according to published culture methods [18]. Amputations were performed on juveniles at 2 weeks post-metamorphosis at the boundary between segment 10 and 11 following a protocol detailed in [19]. *PL10* and *myc* homologs were amplified from mixed stage cDNA by PCR. Amplified fragments were 1192 bp for *PL10* and 964 bp for *myc*. Each fragment was cloned into the pGEM-T_easy_ vector and sequenced for verification (Psomagen Corp, Rockville, Maryland). Gene orthology analysis for *PL10* was previously published [20] and *myc* was published elsewhere [21]. Cloning and probe generation of the following genes were previously published: *runt*, *elav*, *ash*, *Hox3*, *nanos*, *eve2* and *cdx* [19,20,22,23,24]. Whole mount in situ hybridization was performed according to previously published protocols [19,25]. Digoxigenin-labeled riboprobes were generated with the SP6 (*PL10*) or T7 (*myc*) MEGAscript Kit (Ambion, Inc., Austin, TX, USA) and used at a final concentration of 1 ng/µL. Hybridization was performed at 65 °C for 48–72 h. Indirect detection of cell division was performed by labeling cells undergoing DNA synthesis using the Click-iT EdU Alexa Fluor 488 Imaging Kit (Life Technologies C10337), as previously published [19]. Animals were exposed to EdU for 45 min prior to fixation. Labeling with mouse anti-acetylated α-tubulin antibody was performed according to previously published protocols [19]. The antibody was diluted to 1:300 in block solution and incubated for 12–18 h at 4 °C. An Axioskop 2 Mot plus compound microscope (Zeiss), coupled with a SPOT Flex digital camera was used for imaging of specimens for in situ hybridization. Animals processed for immunohistochemistry or EdU labeling were imaged using a Zeiss LSM 710 confocal microscope (Zeiss). Z-stack projections were generated using Fiji (Fiji is Just ImageJ) and images were adjusted in Adobe Photoshop 2021. All figures were created in Adobe Illustrator 2021.

## 3. Results

### 3.1. The Annelid Capitella teleta

**Figure 1:**
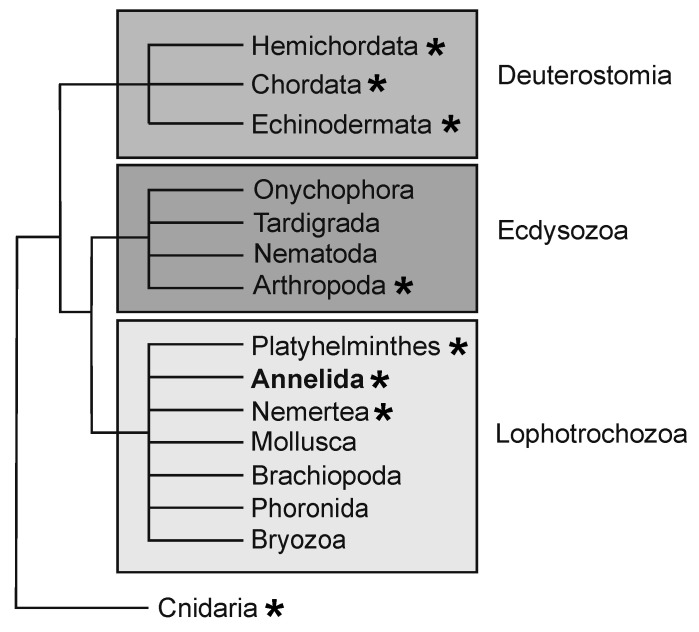
Simplified schematic of bilaterian phylogenetic tree. The three major clades that comprise the Bilateria are depicted by shaded boxes. *Capitella teleta* is a member of the Annelida within the Lophotrochozoa. Taxa with representatives that exhibit whole body or structural regeneration are indicated with an asterisk.

**Figure 2:**
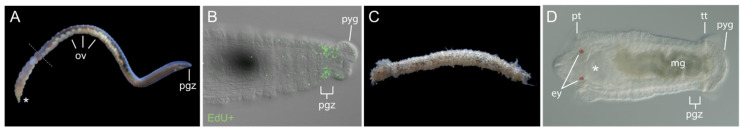
The annelid *Capitella teleta*. Anterior is to the left in all panels. (**A**)Adult *C. teleta* female showing the segmented body organization. Dotted line indicates the boundary between thoracic and abdominal segments. Ovaries present in abdominal segments are visible through the body wall (ov). (**B**) Close up view of the posterior end of a juvenile. Image shows the posterior growth zone (pgz), a localized region of dividing cells that generates segments throughout the lifespan. Green indicates cells in the S-phase of the cell cycle that have incorporated EdU (EdU+). (**C**) Brood tube containing female and embryos. (**D**) Late-stage larva with approximately 13 segments. Shown is a ventral view. Asterisk indicates position of the mouth in panels A and D. Abbreviations: ey, eye; mg, midgut; ov, ovaries; pgz, posterior growth zone; pt, prototroch; pyg, pygidium; tt, telotroch.

*Capitella teleta* [26], previously known as *Capitella* sp. I, is a small annelid worm that has been used as a model for studies in the field of evolution and development [27,28,29]. Phylogenomic analyses place most annelids within one of two clades, the Sedentaria or the Errantia, and *Capitella* is a member of the Sedentaria [27,30]. Similar to other annelids, *Capitella* has complex organ systems, reflecting its phylogenetic position as an annelid and bilaterian (Figure 1). The body of *Capitella* is organized into serially repeated segmental units, with asegmental anterior and posterior termini (Figure 2A). Adults have an indefinite number of segments. Anteriorly, there are nine thoracic segments followed by 45–55 abdominal segments. Similar to many other annelids, *Capitella* generates new segments throughout its life span, undergoing continuous adult development and patterning. New segments are generated near the posterior end of the body from a subterminal region of active cell division known as the posterior growth zone (Figure 2B). Many developmental regulatory genes and germline/multipotency stem cell markers are expressed in the posterior growth zone (Table 1), and this is the case for other annelids that have been examined (reviewed in [4]).

The life cycle and reproductive biology of *C. teleta* are reasonably well characterized. *C. teleta* reproduces sexually, and males and females are easily distinguishable. The male gonads are located within the seventh and eighth segments [31]. Females have highly structured ovaries, which is typical of many polychaetes [32]. In *C. teleta*, ovaries are paired, ventrally positioned within the coelomic cavity, and present in numerous abdominal segments (Figure 2A) [33]. During reproduction, zygotes are laid within a specialized structure made by the female called the brood tube (Figure 2C). The female remains in the tube as the embryos develop. Embryogenesis is characterized by a stereotypical cleavage program called spiral cleavage, in which each blastomere can be uniquely identified [34,35,36]. Embryogenesis is followed by formation of a nonfeeding, swimming larva that emerges from the brood tube approximately nine days after fertilization (Figure 2D). Larvae undergo metamorphosis and become a burrowing juvenile worm. There is substantial subsequent growth, and most segments are generated during juvenile and adult stages. The *C. teleta* life span is approximately 4–5 months in laboratory cultures, and individuals can reproduce multiple times.

### 3.2. Capitella Regeneration Abilities and Favorable Properties for Regeneration Research

*C. teleta* exhibits robust posterior regeneration and is becoming recognized as a study species for annelid regeneration [4,8,29,37]. Its segmented body allows for amputations at precise and reproducible positions. Juveniles are semi-transparent, providing the potential to perform live imaging on amputated worms. Furthermore, regeneration occurs within a practical time frame. By seven days post amputation, multiple new segments appear containing regenerated complex organ systems, such as the digestive tract, centralized nervous system, musculature, kidneys, appendages, coelomic cavity linings, gonads, blood and epidermis. Juveniles and adults can reform segments following transverse amputation at any location posterior of the seventh segment [2,19]. Experimental manipulations demonstrate that regeneration can occur in compromised conditions. Specifically, segments regenerate in the absence of feeding and in the absence of a brain (see below), although fewer segments form [38]. This example underscores the robustness of the posterior regeneration response in *C. teleta*.

**Figure 3:**
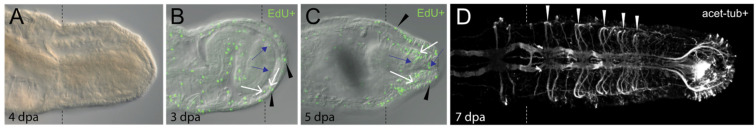
*Capitella* regenerates posterior segments. All panels show posterior end of regenerating juvenile animals in ventral view with anterior to the left. Dotted lines indicate position of amputation site. Animals were amputated between the 10th and 11th segment. (**A**) Posterior regeneration in *Capitella*. (**B**,**C**) Dividing cells are present in ectoderm (black arrowheads), mesoderm (white arrows) and endoderm (blue arrows). Green indicates cells in the S-phase of the cell cycle that have incorporated EdU (EdU+). (**D**) Confocal projection of regenerated ganglia and circumferential peripheral nerves of the central nervous system. White arrowheads indicate pairs of peripheral nerves emanating from newly formed ganglia in the regenerate. There is one ganglion/segment and, thus, five segments have formed in the regenerate. Nerves were labeled with an anti-acetylated tubulin antibody (acet-tub+). Images in B and C are adapted from [38]. Dpa, days post amputation.

The relative order of events that occurs during *C. teleta* regeneration is typical for annelids and other animals that undergo structural or whole-body regeneration [4,19,39]. Following transverse amputation, the wound is rapidly sealed. There is subsequent wound healing through re-epithelialization, presumably by fusion of the cut body wall epidermis. This takes place within 4–6 h in *C. teleta*. Next there is formation of a regeneration blastema, a localized region of undifferentiated cells near the wound site that gives rise to the regenerating tissues. Generation of new tissue occurs through cell proliferation at the wound site. These events are initiated in *C. teleta* approximately 2 days post amputation (dpa). The regenerate grows for several days (Figure 3A) and dividing cells can be observed in all three germ layers [38] (Figure 3B,C). During this time frame, a new posterior growth zone and pygidium form. Subsequently, newly born cells differentiate and undergo morphogenesis to form new tissues that become integrated with pre-existing tissues. Morphogenesis and differentiation are most readily manifest by the appearance of multiple small segments. Segments appear after approximately seven days and are visible by the appearance of new ganglia in the ventral nerve cord (one per segment), segmentally repeated peripheral nerves (one pair per hemisegment) and intersegmental furrows (Figure 3D). Chaetae appear a few days after segment formation. Nascent segments eventually grow to scale of the size of more anterior, pre-existing segments, and there is generation of additional segments from the posterior growth zone.

**Figure 4:**
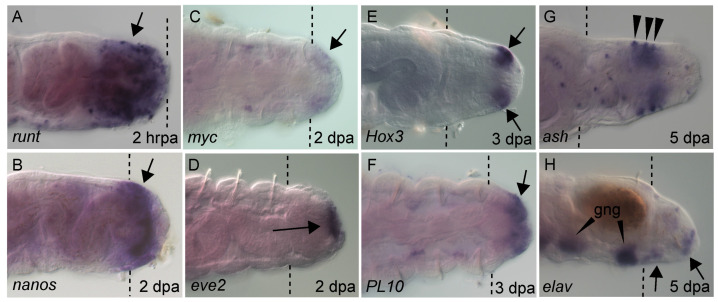
**Gene expression in regenerating tissue.** Whole mount in situ hybridization showing gene expression of developmental regulatory genes at different stages of regeneration. All panels show the posterior end of regenerating animals, with anterior to the left. Purple precipitate indicates mRNA (arrows). Gene name is indicated in bottom left of each panel. Time after amputation is indicated in the bottom right of panel in either hours (hrpa) or days post amputation (dpa). Panels (**B**–**G**) are in ventral view and (**A**,**H**) are in lateral view. (**G**) Punctate expression is *ash* expression associated with the enteric nervous system. (**H**) Ganglia expressing *elav* in pre-existing tissue are indicated (gng). Brown ellipse in panel H is a fecal pellet. Dotted lines indicate amputation site. *Hox3* expression was previously published [19].

Expression of molecular markers at the wound site, in the growing regenerate and during differentiation of the regenerating tissue reflects the sequential events of regeneration. There are available molecular markers for almost all regeneration stages in *Capitella*. Within hours of amputation, the transcription factors *runt* and *post2* are detected at the wound site (Figure 4A) [19]. Homologs of stem markers/multipotent progenitor cells (MPC), such as *vasa*, *nanos*, *piwi1* and *PL10*, are broadly expressed in the 2 dpa regenerate, appearing coincidently with initiation of localized cell division near the wound site (Table 1) (Figure 4B,F) [20,40]. Other stem cell genes such as *myc* are barely detectable (Figure 4C). Around the same time, molecular patterning of the new posterior end becomes visible, exemplified by appearance of localized expression of *eve2* in the anus (Figure 4D). *Eve2* marks the position of the anus in uncut animals [22]. Expression of MPC genes persist as the regenerate grows over several days. Over time, *piwi1*, *vasa* and *nanos* expression domains shift from the posterior end of the regenerate to a subterminal position, likely reflecting establishment of a new posterior growth zone. A similar spatial shift of localized EdU+ cells occurs [19]. As cells of the newly regenerated tissue commit to specific cell fates, the proneural gene *ash* is expressed in the ventral ectoderm and a few isolated cells in the gut (Figure 4G). As cells differentiate in the regenerate, *elav*, a marker for newly differentiated neurons, appears in the nascent ganglia (Figure 4H). The ParaHox gene *caudal* gradually shifts from broad expression in multiple germ layers to restricted expression in the hindgut (see below). *Hox* genes are also expressed in the regenerating tissue (see below). In other annelids that have been examined, similar gene expression patterns in the regenerating tissue have been identified. Most notably, *vasa*, *nanos*, *PL10* and *piwi* homologs are expressed in the blastema of the annelids *Platynereis dumerilli* [17], *Alitta virens* [41] and *Pristina leidyi* [42], and this may be a general molecular feature of annelid regeneration [4,8].

In contrast to its robust posterior regeneration in juveniles and adults, *Capitella* regeneration abilities are not as impressive in other contexts. For example, juveniles and adults are incapable of regenerating their head, although the wound of a posterior body fragment heals and the fragment remains alive for at least a week. The inability to regenerate anterior structures in adults also includes minimal amputations, such as when only the anterior-most part of the body is removed, namely the prostomium or peristomium [2]. Among members of the Capitellidae that have been examined, none exhibit anterior regeneration as adults [2]. On a smaller scale, individual cell types in the head do not appear to reform. For example, eyespots do not reform following their deletion in larvae, even after 1 month [43]. This inability to replace lost tissues extends earlier into embryogenesis, in which deletion of precursors to most tissues in the early-stage embryo results in larvae with missing structures [44]. The time of onset of regeneration abilities during the life cycle is currently unknown. The differences in regeneration potential during the *C. teleta* life cycle provide opportunities to compare regeneration permissive contexts with nonpermissive contexts within a single animal.

Experimental manipulations in *C. teleta* demonstrate that posterior regeneration can occur in compromised conditions. For example, segments can form in the absence of feeding and in the absence of a brain (see below), although the number of segments that form are fewer compared with the number of segments formed on amputated animals that are well fed and have a brain [38]. This example underscores the robustness of the posterior regeneration response in *Capitella*.

There are established resources and techniques available to facilitate regeneration studies in *C. teleta*. It is one of the animal systems with the most available molecular tools within the Spiralia [45]. The availability of a sequenced genome enables molecular, comparative and functional studies. Zygote microinjection and functional genomic techniques such as morpholino knockdown, expression of exogenous RNA and CRISPR/Cas9 mutagenesis are established techniques [29,46,47,48,49]. The stereotypic early embryonic cleavage pattern and availability of a fate map [46] provide opportunities to determine the cellular origins of tissues, including of the germline and regenerating organs. There are molecular and immunohistological markers available for multiple cell types, including of the nervous system, digestive system, eyes, gonads, germline and body wall muscle [20,24,40,43,50,51,52,53,54,55]. Knowledge of the development of these tissues can serve as a framework for comparisons between regeneration and development. A regeneration staging system facilitates comparisons across individuals and studies [19,40]. Because *C. teleta* undergoes sexual reproduction and all stages of the life cycle can be maintained in the laboratory, it is possible to examine changes in regeneration abilities throughout the life cycle. This is particularly relevant for annelids since embryos generally do not replace cells when cells are removed from the early-stage embryo. Data from *C. teleta* can be placed in a phylogenetic framework due to recent considerable progress in determining relationships among annelids [27]. The substantial history of annelid regeneration research on a wide range of species, combined with variation in regeneration abilities across annelids, provide a rich comparative framework for interpreting regeneration data from *Capitella* or other annelids. In summary, *Capitella teleta* is experimentally tractable and has a combination of features and available resources that provide unique opportunities to investigate central biological questions in animal regeneration biology.

In *C. teleta* it is possible to compare regeneration, development and adult growth within a single animal, a strength that sets *C. teleta* apart from some other animal models of regeneration. Such comparisons can help determine the extent of redeployment of developmental genes during regeneration or characterize the relationship between adult segment addition and posterior regeneration. Examination of gene expression patterns indicate that many of the same genes involved in development appear to have similar roles during regeneration. This premise is supported by observations of similar gene expression patterns between regenerating tissue and late-stage larvae, as well as between regenerating tissue and adult posterior growth. It is notable that almost all identified molecular markers expressed in the regenerate of *C. teleta* are also expressed in the posterior growth zone of unamputated animals, as well as during late embryonic and larval development (Table 1). There is a general trend showing very similar expression patterns between a late-stage regenerate and the pattern observed in the posterior growth zone during adult growth. Examples include *Hox* genes, MPC markers (*vasa*, *nanos* and *piwi*), neural markers such as *ash* and *elav* and the gut markers *eve2* and *cdx* (see Table 1 for references). These similarities support previously proposed ideas, specifically, that following wound healing, regeneration of posterior segments is accomplished by a quick transition to a posterior growth mechanism (reviewed in [56]). In contrast, expression patterns during early stages of regeneration tend to be different from patterns observed during larval or adult development. These differences may include expression in a different germ layer or a distinct expression domain. For example, *cdx* is expressed in both the epidermis and gut during early stages of regeneration but has limited expression in the hindgut of uncut animals and at late stages of regeneration (see below). Another example is that of the *Hox* gene *post2*, which initially shows broad expression in the ectoderm of the regenerating tissue, extending from the cut site to the posterior end of the regenerate. At later stages, once new segments appear, *post2* is localized to the newly formed ganglia, a pattern similar to that observed in uncut animals. A substantial difference between embryonic development, adult growth and regeneration is the local environmental context. During regeneration and adult growth, new tissue is patterned and undergoes growth in an environment that contains mature and physiologically active organ systems, whereas in embryonic development most surrounding cells are in a similar state of differentiation. These differences in context likely indicate differences in the initiation of the development/regeneration program. Are similar developmental modules utilized in these different contexts? Both processes need to generate segments with a proper positional identity. Future functional genomic studies are necessary to answer some of these questions, confirm that gene expression patterns accurately indicate gene function and build gene regulatory networks, and that can be compared across these different contexts.

### 3.3. Influence of the Nervous System on Regeneration

**Figure 5:**
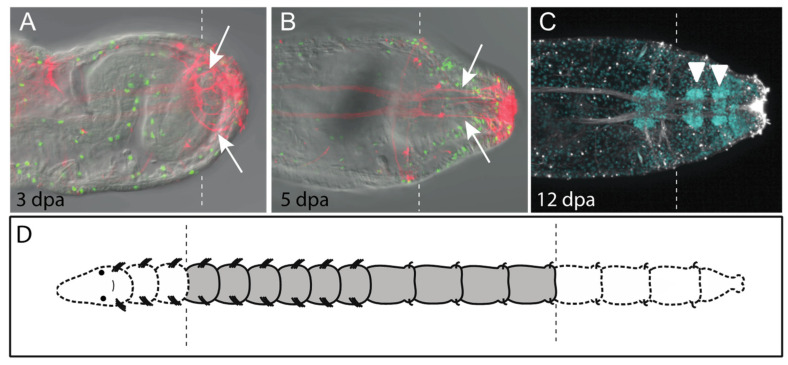
**The central nervous system and regeneration.** All panels are in ventral view with anterior to the left. Vertical dotted lines indicate approximate position of amputation site. (**A**–**C**) are confocal projections. Nerves are shown by acetylated tubulin reactivity (acet-tub+) in (**A**–**C**). (**A**,**B**) Arrows indicate extension of neurites (red) into the regenerating tissue. Green indicates incorporation of EdU. (**C**) Regeneration of posterior segments in the absence of brain and anterior segments. White arrowheads indicate regenerated ganglia. Nerves are indicated in white and nuclei in blue (Hoechst 33342 staining). (**D**) Scheme of double amputations to assess role of brain during posterior regeneration. Dotted areas show regions of body removed during amputation to generate image in C. Images are adapted from [38]. The time following amputation (in days) is indicated in bottom left of panel (dpa).

The nervous system has been shown to be necessary for both vertebrate and invertebrate regeneration, and production of a trophic factor by nerves has been particularly well characterized during amphibian limb regeneration [57]. Observational and experimental evidence from a number of annelids implicate a role of the nervous system in successful regeneration, specifically in the formation and maintenance of the blastema (reviewed in [1,6,7]). These studies provide evidence for a role of the brain as a distant influence and for invasion of neurites into the wound site as a local influence. In *Capitella*, there is a dramatic response of the nervous system near the wound site that occurs within the first few days of amputation [19,38]. At this stage (2 dpa), numerous neurites are dispersed throughout the regenerate, often extending to the posterior end of the animal (Figure 5A). Neurites appear to re-extend directly from the cut end of the ventral nerve cord connectives. These neurites lack the regular arrangement of neurons in the longitudinal and peripheral nerves of unamputated animals. Neurites gradually consolidate into multiple nerves oriented along the anterior-posterior axis as the regenerate grows (Figure 5A). Later, as new tissues differentiate, peripheral nerves appear in the regenerating tissue, extending from newly formed ganglia of the regenerate (Figure 3D). It is currently unknown if the early changes in the nervous system have an instructive role in the regeneration process and, if so, what the molecular nature of such a signal is. Alternatively, the presence of dispersed neurites may simply be part of the regeneration response of the nervous system.

While there is currently no experimental data regarding the role of neurites on subsequent regenerative ability, there is experimental evidence in *C. teleta* about the potential role for the brain in posterior regeneration (Figure 5C,D) [38]. Although the anterior region (e.g., brain) does not regenerate, most individuals form new segments posteriorly in the absence of a brain (Figure 5C). These findings were generated from double amputations in which both ends of the animal were removed, including the brain at the anterior end (Figure 5D). These regenerating animals formed fewer segments compared to amputated animals with an intact anterior end. However, because the anterior amputations also removed the mouth, which resulted in subsequent lack of feeding, it is possible that the reduction in the number of regenerated segments is due to lack of nutritional input. There is variability across annelids in the requirement of a brain for successful regeneration, and animals that can regenerate anteriorly obviously do so in the absence of a brain [6,7,58].

In summary, while it appears that *C. teleta* can regenerate new segments in the absence of a brain, the possibility remains that the nervous system has an important role in *C. teleta* regeneration. It may be that local neural input near the wound is required. Future experimental manipulations are required to implicate a role for the nervous system in *C. teleta* regeneration, potentially from neurites that invade the wound site.

### 3.4. Patterning and Repatterning during Regeneration

**Figure 6:**
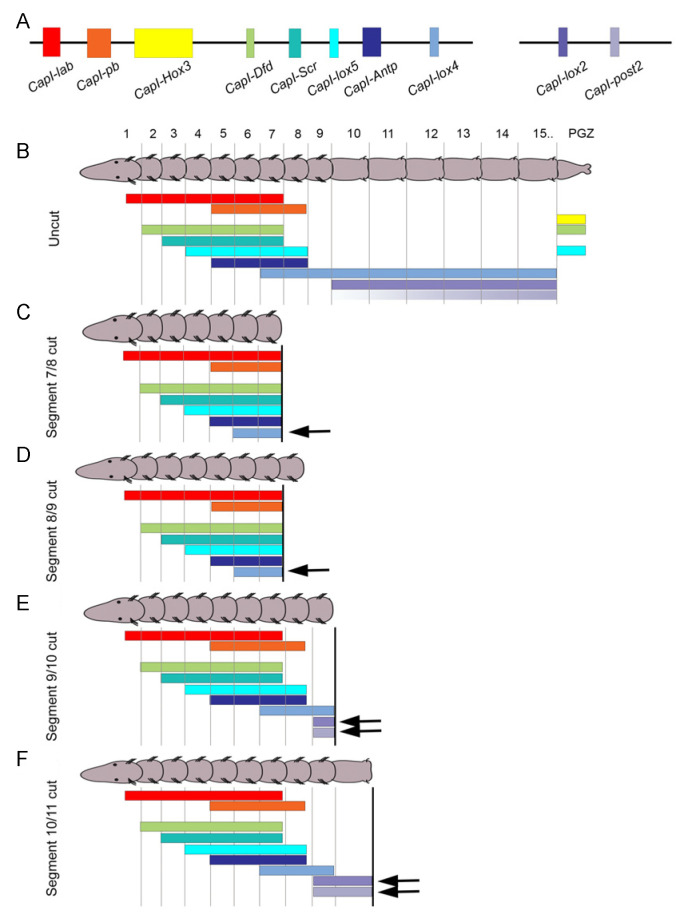
**Effect of amputation location on *Hox* gene expression patterns.** Schematic summarizing *Hox* gene expression patterns in uncut animals and animals amputated at different segmental positions. (**A**) Genomic organization of 10 of the *Capitella Hox* genes present in the *Capitella* genome [59]. Colored rectangles indicate position of coding sequence and black line indicates genomic linkage. (**B**–**F**) Schematics of animals with representation of *Hox* gene expression patterns. Anterior is to the left and the posterior growth zone (pgz) is indicated. Segment number is indicated above animal in B. Colored bars indicate position of *Hox* gene expression along the anterior–posterior axis. Color coding matches that of genes shown in (**A**,**B**). Uncut animals show staggered anterior *Hox* gene expression boundaries. (**C**–**F**) Depiction of expression boundaries 24 hours after amputation at specific segmental positions. Amputations were performed at the boundary between segments 7 and 8 (**C**), 8 and 9 (**D**), 9 and 10 (**E**) and 10 and 11 (**F**). Black arrows indicate examples of shifts in anterior gene expression boundaries in the anterior direction relative to expression boundaries in uncut animals. Adapted from [19].

Regeneration in annelids typically involves remodeling of pre-existing tissues concordant with generation of new tissue through cell division. *Hox* genes are markers of regional identity along the anterior–posterior axis of annelids and can serve as useful markers to track changes in positional identity along the main body axis. The *C. teleta* genome contains 11 *Hox* genes, and 10 of them are linked in the genome on two genomic scaffolds (Figure 6A) [59]. Furthermore, these same 10 genes show staggered, anterior expression boundaries that generally fit the pattern of spatial co-linearity (Figure 6B). Eight of the nine thoracic segments have a unique identity defined by a specific *Hox* code. In contrast, almost all abdominal segments express the same three *Hox* genes. In juveniles, most *Hox* genes show restricted expression in the ganglia of the ventral nerve cord, suggesting that axial positional information is encoded in the nervous system of juveniles [19,59]. This contrasts with the broad ectodermal expression observed in larval stages. Anterior and posterior boundaries of *Hox* gene expression are maintained in the thoracic segments as animals add segments to the posterior end during growth, demonstrating stable positional identity in the nine thoracic segments [19]. *Hox* gene expression was used to follow remodeling of the ‘old’ tissue and examine patterning in the growing regenerate.

Seven of the ten *Hox* genes are expressed in the regenerating tissue in *C. teleta* and each displays a characteristic expression pattern [19]. The temporal onset of expression varies by gene but does not coincide with either the temporal onset during development or with the relative position within the genomic cluster. The earliest *Hox* gene to be expressed regenerating tissue is *Hox3* and it appears as the blastema begins to grow by cell division. *Hox3* has a stable expression pattern as the blastema grows (Figure 4E) and this pattern resembles its expression in the posterior growth zone in uncut animals. The localization of *Hox3* to the posterior growth zone suggests that it does not function in anterior–posterior patterning and that it represents a departure from the spatial co-linearity observed for the other *Hox* genes that are clustered in the genome. *Hox3* shows a similar expression pattern in the annelids *Platynereis dumerilli* and *Alitta virens*, suggesting that it has acquired a new function in posterior growth prior to the split between the Errantia and the Sedentaria [60,61]. Expression for the remaining *Hox* genes in the regenerating tissue becomes detectable during a growth phase and initiation of localized cell division of the blastema. The posterior *Hox* gene *post2* is broadly expressed during the growth phase of the regenerate and gradually shifts to become restricted to the ganglia of the ventral nerve cord [19]. At the time that new tissues form, *Dfd*, *Lox4*, *Lox2* and *post2* are expressed in the nascent ganglia of the regenerate. Other genes such as *Lox5* and *pb* are expressed in small, segmentally repeated cell clusters in the regenerate and may indicate differentiation of segmented structures. Overall, the spatial patterns do not recapitulate the developmental expression patterns observed in larvae. In addition, the spatial patterns do not show co-linearity within the regenerating tissue, which might be expected if the regenerate were an autonomously developing unit, separate from the rest of the body. Once new tissues begin to differentiate (St. V of regeneration), *Hox* gene expression patterns are very similar to those in uncut animals. By inferring function from expression patterns, it appears that in *C. teleta*, *Hox* genes have a role in later stages of regeneration during cell and tissue specification and differentiation. By comparison, *Hox* genes are also expressed in complex patterns in the regenerating tissue of the nereids *P. dumerilii* and *A. virens*, although there are species specific differences in pattern, in timing of expression onset and in which *Hox* gene homologs are expressed [60,61].

Remodeling of the pre-existing tissue is observed as part of the annelid regeneration response in a number of species (reviewed in [4,62]). *Hox* gene expression patterns provide markers to track possible remodeling of segment identity following an amputation event. We initially hypothesized that pre-existing segments would become re-organized to scale following amputation and express the complement of *Hox* genes. Instead, *Hox* genes generally exhibit stable anterior and posterior expression boundaries following amputation at multiple positions along the body length in *C. teleta*, showing general stability of identity in the thoracic segments (Figure 6C–F) [19]. An exception is observed with an anterior shift in expression of three *Hox* genes, *lox4*, *lox2* and *post2* (arrows in Figure 6C–F). A one segment anterior shift occurs when animals are amputated in specific thorax segments and the position that elicits an anterior shift is specific for each of the three genes. The onset of this anteriorly shifted expression also varies among the three genes. In contrast, when amputations are performed further posterior, between the first and second abdominal segment, all *Hox* gene expression patterns in the ganglia of the nervous system are stable, and this stability of expression patterns persists into stages of tissue differentiation in the regenerate (Figure 6F). These data provide evidence of limited neural re-patterning in *Capitella* and show that nervous system identity in the thoracic segments is generally stable, even when the body length is decreased by more than half.

**Figure 7:**
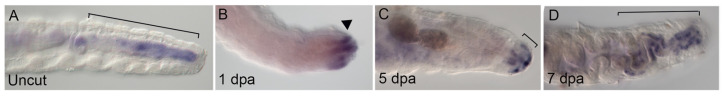
**The ParaHox gene *CapI-cdx* defines new hindgut identity during regeneration.** Animals are amputated anterior to *CapI-cdx* expression domain in the hindgut of uncut animals. *CapI-cdx* expression in the hindgut of uncut animals (**A**). By 1 dpa, novel expression is initiated in pre-existing tissue near the wound site (arrowhead in B) and gradually becomes localized to new gut tissue in the regenerate at 5 and 7 dpa. (**C,D**). Lines in (**A**,**C**,**D**) and arrowhead in (**B**) mark area of expression. A and B are lateral views, C and D are ventral views. Anterior is to the left in all panels. Days post amputation (dpa) is indicated in bottom left corner.

In contrast, examination of the alimentary canal following amputation supports the idea that distinct organs within an animal may differ in the extent to which remodeling occurs. The *C. teleta* gut tube is highly regionalized along its length and includes localized patterns of ciliation [63,64]. Specifically, there are cilia in anterior and posterior portions of the gut that are absent in the middle. When animals are amputated in the body region containing a portion of the gut tube that lacks cilia, cilia appear in the gut approximately 4–5 days following amputation (Figure S2 in [19]). Likewise, the expression profile of *eve2* and the ParaHox gene *CapI-cdx* following amputation indicates re-patterning of a new posterior end (Figure 4D and Figure 7). In uncut juveniles, *CapI-cdx* is localized to the hindgut (Figure 7A) [23]. Following amputation anterior to the *CapI-cdx* expression domain (segment 10/11), novel *CapI-cdx* expression appears at the wound site by 1 dpa, prior to the onset of cell proliferation in the blastema (Figure 7B). At 5 dpa, *CapI-cdx* is expressed in both surface ectoderm and in a small posterior gut domain (Figure 7C). By 7 dpa, *CapI-cdx* expression is restricted to the posterior gut epithelium, similar to its pre-amputation pattern, indicating that the gut has been re-patterned with a posterior gut identity (Figure 7D). In annelids, such remodeling would constitute morphallaxis, although we have not investigated whether the observed changes can occur in the absence of cell division. The general stability of *Hox* gene expression patterns and dynamic expression of gut markers indicate different responses of the gut and nervous system to amputation in *Capitella*. These results support the hypothesis that distinct organs may show differing remodeling responses in annelids.

Tissue remodeling during regeneration has been documented in several annelids. For example, gut remodeling occurs during anterior and posterior regeneration in *Enchytraeus japonensis*, *Pristina leidyi* and *Alitta virens* [65,66,67]. In serpulids and sabellids (fan worms), ectodermal remodeling occurs during anterior regeneration [15,68]. Specifically, these animals have patterns of chaetae or bristles positioned asymmetrically along the dorsal–ventral axis. Normally, the thoracic and abdominal segments exhibit inverse patterns in their chaetal arrangement. During anterior regeneration from abdominal segments, a shift occurs from abdominal to thoracic segment identity and a concordant inversion in the arrangement of distinct chaetal types. Examination of multiple tissues within a single species in additional annelids will provide a more complete picture of tissue remodeling during regeneration and whether distinct tissues may respond independently to amputation. Studies of tissue remodeling in annelids provide opportunities to investigate in vivo cellular reprogramming.

### 3.5. Identification of a Putative Multipotent Stem Cell Niche

**Figure 8:**
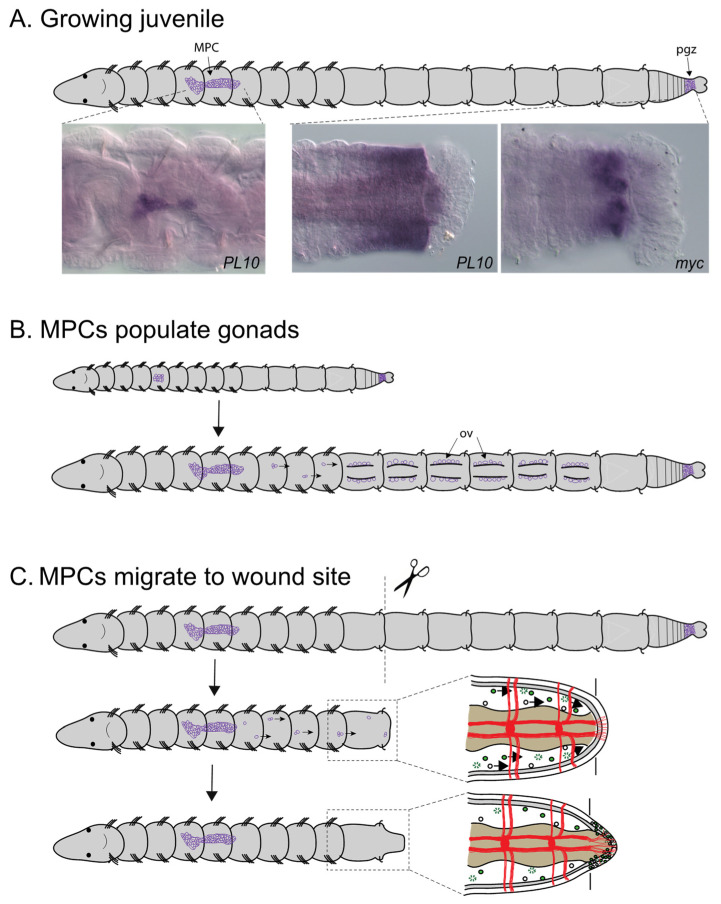
**Hypothesized roles for MPC cluster in *Capitella*.** All images are oriented with anterior to the left and in ventral view. (**A**) Schematic of juvenile worm showing location of MPC cluster (MPC) and posterior growth zone (pgz), two domains that express somatic/germline stem cell markers (purple). High magnification inset images show gene expression of *PL10* in the MPC cluster and posterior growth zone and *myc* in the posterior growth zone. Purple precipitate indicates mRNA localization using whole mount in situ hybridization. Dotted lines indicate enlarged area of body shown for gene expression patterns. (**B**) Working model for role of MPCs in populating gonads with germ cells. As the ovaries mature, cells from the MPC cluster migrate (arrows in bottom schematic) several segments to reach the ovaries, which are located in the abdominal segments. (**C**) Working model of MPC response during regeneration. Amputation initiates a signal from the wound site. Cells from the MPC respond by migrating towards the wound site (arrows in center images). Migrating and locally derived cells populate the blastema and contribute to its growth (bottom images). Top, uncut animal; middle, animal soon after amputation; bottom, amputated animal after blastema has formed. Dotted line boxes indicate area of detail shown to right for middle and bottom images. Top schematic, young juvenile worm. Bottom schematic, older individual with ovaries that contain immature oocytes. Abbreviations: ov, ovary; MPC, multipotent stem cell cluster; pgz, posterior growth zone.

Expression analysis of the stem cell/germline markers *vasa* and *nanos* in *C. teleta* adults led to identification of a cluster of putative multipotent progenitor cells (MPC) [20]. In adults, this cluster contains approximately 75 cells that have an undifferentiated morphology and a large nuclear to cytoplasmic ratio. It is suspended by mesenteries in the coelomic cavity of segment 5, ventral of the gut tube (Figure 8A). Unlike many other structures in the body, this cluster is not segmentally repeated. *Piwi1*, *piwi2*, *PL10* and *myc* homologs were later found to also be expressed in this cell cluster [38,40] (Figure 8A). *Piwi1*, *piwi2* and *PL10* are broadly expressed by cells within the cluster and show very similar expression patterns to each other. In contrast, *myc* is expressed in a small subset of cells in the cluster, suggesting molecular heterogeneity among cells in the cluster [38]. This expression profile is characteristic of a ‘germline multipotency program’ [69].

Development of the MPC cluster has been characterized from expression data of fixed tissue. In mid-larval stages, there are small bilateral cell clusters each containing 2–5 cells of *vasa*+, *piwi1*+, *piwi2*+ and *nanos*+ cells in the mesoderm of midbody segments [20,40]. Fate mapping studies using intracellular lineage tracers have identified cells with a similar number, position and morphology that arise from the blastomere 4d in early-stage embryos [46]. In late larval stages, there is a single cluster of 8–20 cells located at the midline. Following metamorphosis, the number of cells in the MPC cluster gradually increases. In one-week-old juveniles the cluster contains 20–55 cells and there are approximately 75 cells in reproductive adults [40,53]. Dividing cells can occasionally be observed within the cluster in juvenile stages [38]. The number of cells in the cluster remains relatively constant, even after amputation in juveniles [38]. Use of long-term lineage tracers in living material is required to connect these fixed tissue observations.

The MPC cluster may function as a primordial germ cell (PGC) niche in *C. teleta*. During development, prior to the formation of gonads, the cluster may serve as an intermediate target for PGCs in larvae and young juveniles [40]. The presence of an intermediate target for PGCs during development has previously been proposed for *P. dumerilli* [70]. At the time gonads become visible, *piwi*+ and *vasa*+ cells are also present in the coelomic cavity posterior to segment 6, leading to the proposal that cells migrate from this structure to populate the gonads, where they undergo gametogenesis (Figure 8B) [40,53]. In females, the distance to the segmentally repeated paired ovaries is a minimum of four segments away and, in males, the cluster is located one to two segments from the male gonad. The MPC cluster is also maintained in adults and may function as a cellular source to populate ovaries at later stages. For example, it may provide a critical source of primordial germ cells in regenerating ovaries [40]. Second, since environmental stimuli can induce males to become hermaphrodites, it may also serve as a cell source to populate newly forming ovaries. Finally, since adults can reproduce multiple times, cells from this cluster may populate the gonads to provide gametes for subsequent matings. These observations support the idea that *Capitella* maintains a primordial germ cell niche throughout its life span.

Additional observations suggests that the MPC cluster also contains somatic stem cells. In *C. teleta*, removal of the MPC cluster through amputation results in loss of regeneration of posterior segments [38], providing functional evidence for a role of this cluster in conferring successful regeneration of somatic tissue. The observation of *vasa*-positive cells in the coelomic cavity between the MPC cluster and the wound site suggests that cells from this cluster migrate to the wound site from segment 5. In addition, there is evidence that proliferating cells migrate to the wound site (Figure 8C). Data to support this observation utilized an indirect cell tracking method in living animals, specifically the incorporation of the modified nucleotide EdU into dividing cells prior to amputation [38]. Some of the EdU+ cells that became incorporated into the blastema subsequently divided as shown by later incorporation of a second modified nucleotide (BrdU). There may also be a contribution to newly formed tissue from local cellular sources as is reported to be the case for other annelids [1,7,71]. Expression of *vasa*-expressing cells with neoblast-like morphology has also been observed in the coelomic cavity of *E. japonensis* [72] and of *piwi*-expressing cells in the coelomic cavity of *P. leidyi* [73]. Since *vasa*, *nanos*, *piwi1, piwi2*, *PL10* and *myc* are expressed in the MPC cluster, gonads and the posterior growth zone of *C. teleta* (Table 1) (Figure 8A), it is likely that these genes have a dual role in both somatic and germline cells. *Vasa*, *nanos*, *piwi* and *PL10* homologs are expressed in both the posterior growth zone and gonads in other annelids (reviewed in [4]). Morphological similarities between stem cells and germ cells have long been noted in annelids [12,13].

Such an expression profile in both somatic and germ cells has been proposed to reflect an evolutionary link between somatic and germline stem cells in many animals, including in early branching metazoans (reviewed in [69]). Further, observations based on gene expression patterns in fixed tissue, indirect cell labeling and experimental manipulations have led to generation of testable hypotheses concerning cellular origins of regenerating tissue and the possible contribution of cells from the MPC cluster. Taken together, cells in the MPC cluster may serve as a source of both germline and somatic stem cells and, therefore, represent a multipotent cell population.

### 3.6. Regeneration of the Germline and Gonads

Regeneration abilities vary during the life cycle of *C. teleta*. During early embryogenesis, removal of single precursor cells typically results in larvae with missing structures that normally arise from that cell [44]. This general inability to replace parts removed early in the life cycle contrasts with the robust regeneration of posterior segments in juveniles and adults. It is currently unknown exactly when in the life cycle this shift in regeneration potential occurs and if this transition is abrupt or occurs gradually.

One exception to the general inability to replace larval structures following removal of embryonic precursors in *C. teleta* is the germline. The germline is the developmental precursor of the gametes and is essential for successful sexual reproduction. In many animals, the germline is segregated from the somatic tissue early in embryonic development [74] and removal of the germline from adults or removal of germline precursors from embryos results in sterile animals in some animals [75,76,77,78,79,80,81,82]. 

*Capitella* has several advantages for analysis of germline reformation. First, it has a stereotypic cleavage program. Individual blastomeres in the early-stage embryo can be uniquely identified [36]. Second, the embryonic origins of almost all larval tissues have been determined by fate mapping using intracellular lineage tracers, including the origins of the germline [46]. Lineage tracing plus gene expression studies are consistent with an origin of the germline by the cell 4d [20,40,46]. Third, the germline and mesoderm do not arise from same precursor cell, allowing for experimental manipulation of the germline without disrupting formation of other critical mesodermal structures such as body wall muscle. In addition, it is straightforward to identify gonads and gametes in live animals and perform successful matings in the laboratory.

Experimental manipulations of early-stage *C. teleta* embryos demonstrate an ability to replace the germline [53,83] (reviewed in Wessel et al., 2020). All juveniles resulting from embryonic deletion of germline precursors have germline cells and all mature adults have reproductive structures. Furthermore, all adults with reproductive structures are fertile and produce morphologically normal, swimming larvae. In contrast, targeted deletion of the germline precursor in the early embryo results in a high proportion of larvae that lack *vasa*, *nanos* and *piwi1* expression, markers of germline and male and female germ cells in *C. teleta*. Only a small proportion of larvae have germline precursors (13%, *n* = 89), indicative of limited germline regeneration. The dramatic and statistically significant difference in ability to regenerate the germline between larval and adult stages suggests that there may be two distinct compensation events: a regulative event during larval stages and a stem cell transition after metamorphosis when animals can undergo substantial body regeneration. The cellular origins of the new germline are not yet definitively known, although precursors of the trunk mesoderm are obvious candidates since the germline originates from mesoderm in many animals. The germline reforms following deletion of the germline plus D quadrant trunk mesoderm (blastomere 2D). This result indicates that the new germline arises from either ectoderm or mesoderm outside of the D quadrant (blastomere 3c); either way, this is an unprecedented finding in spiralian animals. The finding that *Capitella* can regenerate its germline following its removal in the embryo is an example of cellular reprogramming from somatic to germline cells.

Although the ability to reform germline from somatic cells has not been widely reported in annelids, there is indication of complex germline origins that arise post-embryonically during induction of the sexual phase in *P. leidyi* [42]. Outside of annelids, it has been shown that the germline can reform following its removal in the flatworms *Macrostomum lignano*, *Schmidtea mediterranea* and *Dugesia japonica*, and in the ascidians *Ciona intestinalis* and *Botryllus primgenus* [84,85,86,87,88]. It is notable that these examples include animals with substantial adult regeneration abilities and suggests a link between somatic and germline regeneration.

Female gonads can also regenerate in *C. teleta* [40]. Ovaries form in regenerating posterior segments of females and the regenerated ovaries become populated by *piwi+* cells. The cellular source of oocytes that appear in regenerating ovaries is not currently known. Possible alternatives include that regenerated oocytes arise de novo from a local cellular source or that germline stem cells migrate from a distant site. Regeneration of male gonads has not been examined in *C. teleta*. An ability to regenerate its gonads and replace lost germline through reprogramming are manifestations of resiliency in the reproductive abilities of *C. teleta*. Another example is illustrated by examination of the impact of amputation on fecundity. The reproductive output of females is minimally affected by amputation and regeneration when adult females are amputated between the 22–25th abdominal segment [89].

The ability of *Capitella* to replace its ovaries following amputation is not surprising since a number of annelids can regenerate their gonads during anterior or posterior regeneration. Examples include *P. leidyi* [73], *E. japonensis* [90] and *Lumbricillus lineatus* [91,92]. 

## 4. Conclusions

Despite their long history serving as material for regeneration studies, annelids have lagged behind current animal models of regeneration. This is possibly due to a lack of functional tools for studying post-embryonic stages and an inability to routinely culture species that have been historically used for regeneration research. *Capitella* is one of the few annelid species with robust regenerative abilities for which there are also functional genomic tools. The ability to manipulate gene function will be critical for building an understanding of the molecular mechanisms and gene regulatory networks underlying annelid regeneration. Future efforts can extend available embryonic morpholino knockdown and CRISPR/Cas9 mutagenesis techniques to studies of post-embryonic development and regeneration by establishing direct delivery methods into juveniles and adults. Interpretations of data generated for *C. teleta* can be compared with previous annelid studies, particularly with annelids that have different regeneration abilities. Development of these experimental approaches can then be modified for other annelid species, such that comparisons can be framed within the context of available robust annelid phylogenies. Building an understanding of annelid regeneration can serve to contextualize regeneration in other regenerating lophotrochozoans, such as planarians, mollusks and nemerteans, as well as in more broad comparisons among regenerating animals.

Observations based on fixed preparations and gene expression patterns during posterior regeneration in *C. teleta* have led to generation of testable hypotheses concerning cellular origins of regenerating tissue and the germline, as well as the possible contribution of cells from the MPC cluster. Live cell imaging coupled with long term lineage tracing tools will facilitate determination of the cellular origin of regenerating tissue. Specifically, such methods will help distinguish between local cellular sources or distant migratory stem cells (such as the MPC cluster) of the newly forming tissue or contributions from both cell sources. There are additional questions that can be addressed using lineage tracers. For example, as the number of cells in the MPC cluster increases in juveniles and young adults, are all cells in the MPC cluster descendants from a single precursor lineage or do they arise from multiple sources? Does the MPC cluster serve as a source of pluripotent cells that can generate both germline and somatic cells? Questions such as these are fundamental questions in regeneration biology [10,93] and highlight the strength of utilizing the tools available in *C. teleta* for regeneration studies.

Another area to which *C. teleta* regeneration studies can potentially contribute is in building an understanding of the relationship between regeneration and embryogenesis. Are the same molecular players involved during adult growth and regeneration or is there limited overlap? If there are shared genes involved in the two processes, are they wired into the same gene regulatory networks (GRNs) that utilize distinct signals to initiate the process or are there distinct GRNs? Since regeneration abilities differ in different contexts (anterior vs. posterior regeneration, life cycle stage, position along the body), studies in *Capitella* have the potential to uncover permissive versus non-permissive contexts for regeneration.

## Figures and Tables

**Table 1:** Comparison of gene expression between regeneration, growth and development.

Gene Name		Tissue	Expression *			Reference
	Regenerate	pgz	MPC cluster	gonads	larvae	
***piwi1***	+	+	+	+	+	[40]
***piwi2***	?	+	+	+	+	[40]
***nanos***	+	+	+	+	+	[20], this paper (Figure 4)
***vasa***	+	+	+	+	+	[20,38]
***PL10***	+	+	+	+	+	This paper (Figure 4, Figure 8)
***myc***	-/+	+	+	+	+	[38], this paper (Figure 4)
***eve2***	+	+	-	-	+	[22], this paper (Figure 4)
***cdx***	+	-	-	-	+	[23], this paper (Figure 7)
***Hox3***	+	+	-	-	+	[19]
***post2***	+	+	-	-	+	[19]
***runt***	+	-/+	-	-	+	[22], this paper (Figure 4)

*, determined by whole mount in situ hybridization; -/+, indicates either weak or limited expression; MPC, multipotent progenitor cells; pgz, posterior growth zone.
